# Review of seasonal influenza in Canada: Burden of disease and the cost-effectiveness of quadrivalent inactivated influenza vaccines

**DOI:** 10.1080/21645515.2016.1251537

**Published:** 2016-11-18

**Authors:** Edward W. Thommes, Morgan Kruse, Michele Kohli, Rohita Sharma, Stephen G. Noorduyn

**Affiliations:** a GSK, Health Economics and Outcomes Research, Mississauga, Ontario, Canada; b Department of Mathematics and Statistics, University of Guelph, Guelph, Ontario, Canada; c Optum, Health Economics and Outcomes Research, San Jose, CA, USA; d Optum, Health Economics and Outcomes Research, Burlington, Ontario, Canada; e GSK, Medical Affairs, Mississauga, Ontario, Canada; f GSK, Health Economics and Outcomes Research, Mississauga, Ontario, Canada

**Keywords:** burden, costs, epidemiology, hospitalization, influenza, influenza vaccine, lineage-mismatch, quadrivalent

## Abstract

In the 2015/16 influenza season, the Canadian National Advisory Committee on Immunization (NACI) recommended vaccination with quadrivalent inactivated influenza vaccine (QIV) for infants aged 6–23 months and trivalent inactivated influenza vaccines (TIVs) or QIVs in adults. The objective of this review (GSK study identifier: HO-13-14054) is to examine the epidemiology and disease burden of influenza in Canada and the economic benefits of vaccination. To inform this review, we performed a systematic literature search of relevant Canadian literature and National surveillance data. Influenza B viruses from phylogenetically-distinct lineages (B/Yamagata and B/Victoria) co-circulate in Canada, and are an important cause of influenza complications. Modeling studies, including those postdating the search suggest that switching from TIV to QIV in Canada reduces the burden of influenza and would likely be cost-effective. However, more robust real-world outcomes data is required to inform health policy decision makers on appropriate influenza vaccination strategies for Canada.

## Introduction

Many countries have implemented influenza vaccination as targeted programs for people at high risk for influenza-related complications, while some countries such as Australia, Canada, and the United States (US) recommend universal vaccination against seasonal influenza.^1-3^ The aim of influenza vaccination in Canada is to prevent serious outcomes such as hospitalization and death. As such, the National Advisory Committee on Immunization (NACI) stresses the need for vaccination, particularly for those people at high risk for influenza-related complications, including infants 6 to 59 months of age, adults 65 y and older, and people with medical co-morbidities.^4^

Two phylogenetically-distinct influenza B lineages (B/Yamagata and B/Victoria) emerged globally in the early 1980s, and have co-circulated in the US since 2000.^5^ Between 2000 and 2010, the influenza B strain in the seasonal trivalent influenza vaccine (TIV) did not match the prevalent B lineage in around half of the European and North American influenza seasons.^5^ As a result, the World Health Organization (WHO) recommended the inclusion of an influenza strain from each of the influenza B lineages for the 2012/13 influenza season onwards.^6^ Traditional TIVs include either B/Victoria or B/Yamagata whereas quadrivalent influenza vaccines (QIVs) include strains from both lineages. Thus the new QIVs are expected to reduce the burden of influenza B disease,^7,8^ and this is supported by the available evidence. The first efficacy study of a QIV was a randomized, observer-blinded assessment of *FluLaval*™ *TETRA* in children from 3 to 8 y of age.^9^ The study showed that the QIV versus control reduced real-time polymerase chain reaction (RT-PCR)-confirmed influenza A and B cases by 59.3% and reduced moderate-to-severe influenza cases by 74.2%.^9^ A recent meta-analysis of randomized controlled trials of QIVs found that inactivated QIVs have equivalent immunogenicity and efficacy against the shared 3 strains included in TIVs, with superior immunogenicity against the B lineage not included in TIV.^10^

In Canada, a number of influenza vaccines are currently licensed for use. These include 6 inactivated TIVs; split virus or subunit TIVs for intramuscular injection (*Agriflu* [Seqirus], *Fluviral*™ [GSK], *Fluzone, Vaxigrip* [Sanofi Pasteur], and *Influvac* [BGP Pharma ULC], and an MF59-adjuvanted TIV (*Fluad* [Seqirus]).^1^ Three QIVs are also available; a live attenuated influenza vaccine (LAIV) for intranasal administration (*FluMist Quadrivalent* [MedImmune/AstraZeneca]) and 2 split-virion, inactivated QIVs (*FluLaval*™ *TETRA* [GSK] and *Fluzone Quadrivalent* [Sanofi Pasteur]).^1^ TIVs and QIVs are recommended for use in healthy adults and those with chronic health problems from 18 y of age.^1^ NACI preferentially recommends QIV for use in infants from 6 to 23 months of age and LAIV for children 2 to 6 y of age. In the 2015/16 season, NACI offered no preference between TIV, QIV, or MF59-adjuvanted TIV for those 65 y of age and older.^1^

The principal objectives of this literature review are to examine the epidemiology and disease burden of influenza in Canada and the economic benefits of vaccination. To facilitate this, we performed a comprehensive literature review using a detailed search strategy conducted to identify original research of influenza in the general population in a Canadian setting (GSK study identifier: HO-13-14054).

## Search strategy

Search strings were developed to identify potentially relevant sources based on incidence, prevalence, morbidity and mortality of influenza in Canada, along with costs and cost-effectiveness or cost-utility analyses. With these, searches were performed in PubMed, EMBASE and the Cochrane databases for the period from January 2002 to December 2013. The detailed search strategies for PubMed are described in Tables S1, S2 and S3. Search criteria used to identify studies of interest were broad to include epidemiological, clinical and cost-effectiveness studies reporting on influenza in the general population (not pregnant women, diabetics, or HIV+ [human immunodeficiency virus-positive] patients) in the Canadian setting. Identified literature was assessed independently by 2 reviewers for relevance, with any disagreements resolved by discussion and consensus. From these searches, we identified a total of 1,580 citations in the search of electronic databases. Following article appraisal, 64 articles were selected to inform this review. Of these, 27 studies contained relevant data on disease epidemiology, 42 studies on the burden of disease and costs of illness, and 7 studies on the economic evaluation of vaccines. An overview of the search results and selection is presented in Figure S1. We also searched ‘gray’ literature, including annual reports from Canada's influenza surveillance through the Canadian FluWatch surveillance system for the influenza seasons beginning 1999/00 up until 2009/10, including final weekly reports for 2010/11, 2011/12, and 2012/13, along with NACI statements describing vaccine match-mismatch data.

## Canadian surveillance data

**Figure 1.**
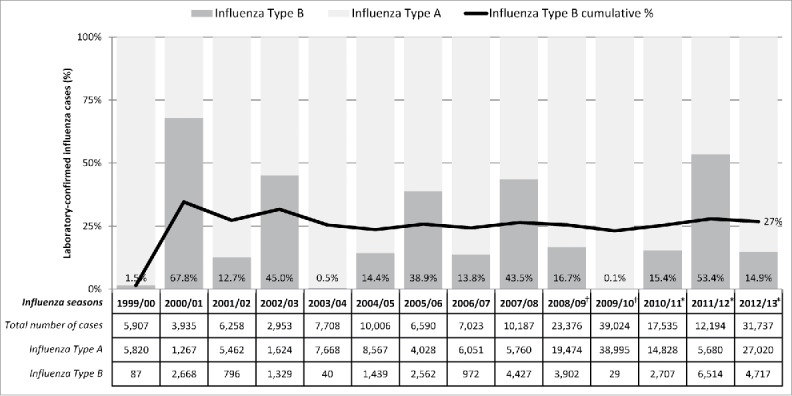
FluWatch surveillance system case-by-case data of laboratory confirmed influenza by season and virus subtype. Note: Data obtained from The Canadian FluWatch surveillance system unless otherwise indicated;^11-22^ Data for 2008/09 and 2009/10 pandemic seasons have not been included for calculation of average influenza B incidence across seasons. ^†^Data are based on aggregate cases for these years only. More sentinel laboratories report aggregate data then detailed data, therefore the case counts are expected to be higher. *Data obtained from FluWatch final cumulative weekly report in absence of a full annual report.^20-22^

Using incidence of laboratory-confirmed cases of influenza taken from the Canadian FluWatch surveillance system for seasons from 1999/00 to 2012/13, the mildest season was 2002/03 (2,953 cases) and the most severe season was 2012/13 (31,737 cases).^11-22^ Data for 2012/13 season were also obtained from NACI statement on seasonal influenza vaccine for 2013/14.^23^ Influenza A was the dominant subtype circulating in all but 2 seasons (2000/01 and 2011/12). Influenza B subtype was reported in fewer than 20% of cases for most seasons, apart from 2000/01, 2002/03, 2005/06, 2007/08, and 2011/12 (68%, 45%, 39%, 44%, and 53% respectively) (Fig. 1). Excluding the pandemic seasons (2008/09 and 2009/10), influenza B has represented an average of 26% to 34% of all laboratory-confirmed influenza cases from 2000/01 to 2012/13, depending on whether one uses, respectively, data from Canada's Respiratory Virus Detection Surveillance System (RVDSS), or the National Microbiology Laboratory (NML) of the Public Health Agency of Canada (PHAC) [internal GSK analysis of publicly-available FluWatch data]. In 14 Canadian influenza seasons for which data was available, 3 seasons showed only one dominant B strain whereas 2 or 3 different strains were reported in the remaining seasons (Table 1).^11-22^ Available FluWatch data show that in the seasons from 1999/00 to 2009/10, most cases were observed in children aged <5 years or adults aged ≥65 years (Table 2).^11-19^ In all seasons, the proportion of seasonal influenza cases in children aged <5 years was >10%. In adults aged ≥65 years, the proportion of cases was particularly high in the 1999/00 season (42%), and in the 2004/05 season (51% and 22% for Influenza A and B, respectively). During three seasons, <10% of cases were elderly people, including the pandemic seasons (2008/09 and 2009/10).

**Table 1.** FluWatch surveillance system reports of influenza B strains in Canada during influenza seasons from 1999 to 2012.

	Influenza season starting:													
	1999	2000^†^	2001	2002	2003	2004	2005	2006	2007	2008	2009	2010^*^	2011^*^	2012^*^
Total cases of influenza B	43	254	152	128	40	214	472	119	673	570	7	586	965	602
B/Victoria (Yamanashi-like)	—	100%	—	—	—	—	—	—	—	—	—	—	—	—
B/Victoria (Beijing-like)	100%	—	—	—	—	—	—	—	—	—	—	—	—	—
B/Yamagata (Sichuan-like)	—	—	3%	—	83%	—	—	—	—	—	—	—	—	—
B/Victoria (Hong Kong-like)	—	—	97%	100%	18%	21%	29%	—	—	—	—	—	—	—
B/Yamagata (Shanghai-like)	—	—	—	—	—	79%	1%	90%	—	—	—	—	—	—
B/Victoria (Malaysia-like)	—	—	—	—	—	—	70%	10%	3%	66%	14%	—	—	—
B/Yamagata (Florida-like)	—	—	—	—	—	—	—	—	97%	2%	14%	—	—	—
B/Victoria (Brisbane-like)	—	—	—	—	—	—	—	—	—	32%	71%	95%	48%	23%
B/Yamagata (Wisconsin-like)	—	—	—	—	—	—	—	—	—	—	—	5%	53%	77%

Note: Data obtained from The Canadian FluWatch surveillance system.^11-22^

† There was 1 case of B/Beijing-like influenza in 2000/01 (0.4% of cases). Data not shown due to rounding.

* Data obtained from FluWatch final cumulative weekly report in absence of a full annual report.^20-22^

**Table 2.** Influenza case distribution by age groups and viral subtype in Canada.

Year^†^	<5 years	5–9 years	10–14 years	15–24 years	25–44 years	45–64 years	65+ years	Age unknown
1999/00 (N = 5,907)^‡^	16.2%	3.4%	1.9%	4.4%	12.9%	14.8%	42.2%	4.1%
2000/01 (N = 3,935)^‡^	24.0%	13.0%	8.0%	16.0%	21.0%	8.0%	8.0%	2.0%
2001/02 (N = 6,258)^‡^	28.0%	6.0%	8.0%	9.0%	11.0%	8.0%	27.0%	3.0%
2002/03 (N = 2,953)^‡^	NR	NR	NR	NR	NR	NR	NR	NR
2003/04 (N = 7,708)^‡^	33.0%	5.0%	5.0%	11.0%	11.0%	9.0%	25.0%	1.0%
2004/05 (N = 10,006)								
Influenza A (n = 8,567)	11.0%	3.0%	3.0%	5.0%	12.0%	13.0%	51.0%	2.0%
Influenza B (n = 1,439)	18.0%	12.0%	5.0%	7.0%	19.0%	13.0%	22.0%	4.0%
2005/06 (N = 6,590)								
Influenza A (n = 4,028)	23.0%	9.0%	5.0%	8.0%	18.0%	12.0%	25.0%	0.0%
Influenza B (n = 2,562)	22.0%	20.0%	19.0%	20.0%	9.0%	6.0%	4.0%	0.0%
2006/07 (N = 7,023)								
Influenza A (n = 6,051)	23.0%	11.0%	7.0%	5.0%	22.0%	11.0%	20.0%	2.0%
Influenza B (n = 972)	19.0%	10.0%	3.0%	0.0%	24.0%	21.0%	23.0%	0.0%
2007/08 (N = 10,187)								
Influenza A (n = 5,760)	24.0%	6.0%	4.0%	11.0%	22.0%	14.0%	19.0%	0.0%
Influenza B (n = 4,427)	17.0%	11.0%	4.0%	4.0%	21.0%	17.0%	26.0%	0.0%
2008/09 (N = 23,376)^‡^	16.0%	11.0%	11.0%	18.0%	22.0%	14.0%	8.0%	0.0%
2009/10 (N = 39,042)^‡^	16.0%	14.0%	12.0%	17.0%	21.0%	16.0%	4.0%	0.0%
2012/13^*^ (N = 23,293)		5–19 years		20–44 years				
Influenza A (n = 20,262)	13.1%	7.2%		15.5%		17.0%	47.3%	0.0%
Influenza B (n = 3,031)	20.4%	26.2%		16.9%		16.8%	19.7%	0.0%

Note: NR, not reported; Data obtained from The Canadian FluWatch surveillance system unless otherwise indicated.^11-19^

† Data were not available in the available FluWatch cumulative weekly reports for 2010/11, 2011/12 and 2012/13 seasons.

‡ Data by viral subtype were not available.

* Data were obtained from NACI statement on seasonal influenza vaccine for 2013/14.^23^

## Influenza attack rates

A number of studies report seasonal influenza attack rates from various settings, including 3 which report the incidence of A(H1N1)pdm09. Most studies were conducted in institutional settings rather than the community and contained limited data specific to influenza B (Table S4).^24-33^ In one study of people dwelling in long-term care facilities, the incidence of influenza B was higher in the residents (6,400 per 100,000 population) than in the staff (3,100 per 100,000 population).^30^ In another study of patients hospitalized with any influenza during the 2007/08 season, the incidence of influenza B was 23 per 1,000 patients.^31^

## Co-morbidities and mortality

The most common co-morbidities associated with seasonal influenza disease in Canadian children were pneumonia and asthma, and the most common co-morbidities in adults were identified as cardiac conditions and respiratory disorders such as pneumonia and chronic lung disease (Table S5).^24,26,27,29,32,34-37^

Between 2008 and 2012, Statistics Canada reported an annual death toll between 5,106 and 5,826 due to influenza and pneumonia (ICD-10 codes J09 – J18).^38^ Many studies have reported influenza-related mortality data in Canada, describing either the probability of death, or formal mortality rates, or both measures (Table S6).^24,26-29,34-36,39-61^ In these, mortality varied widely (0–27%) depending on the population, strain type, and pandemic or non-pandemic seasons.

## General practitioner (GP) and emergency room (ER) visits

GP visit data for influenza infection were reported in 3 studies.^28,33,37^ In a study of an elderly population in a nursing home with seasonal influenza, 66% of those with influenza received visits from a physician,^28^ and a telephone survey of 1,009 Canadian households reported a rate of 0.7 GP visits related to influenza-like illness (ILI) per household.^33^ Information on ER visits was provided by one study which reported that the rate of influenza-attributable ER visits during a non-pandemic influenza season was 460 per 100,000 population.^62^

## Antibiotics and antivirals

From the available data, around 60% of patients with seasonal influenza are reported to have received antibiotics (Table 3).^24,27-29,34-36,39,40,60,61^ In children, the probability of receiving antivirals during seasonal years ranged from 6.6%^61^ to 13%,^63^ while the probability in adults ranged from 29%^26^ to 79.9%.^36^ However, in contrast with seasonal influenza, antiviral use during pandemic years reached 84%^44^ in children and 94.4% in adults.^49^

**Table 3.** Burden of seasonal influenza in Canada expressed as probabilities of outcomes.

Source	Disease	Population	Age (years)	p(antiviral|flu)	p(antibiotics|flu)	p(hosp|flu)	p(ICU|flu)	p(MV|flu)
Fanella 2011^63^	Seasonal influenza A or B	—	Under 18 years; median = 1.08	13.0%	—	—	26.1%	66.0%
Moore 2006^60^	Influenza	—	Children; median = 1.7	7.0%	77.0%	—	12.0%	6.2%
Tran 2012^34^	Seasonal influenza A	Hospitalized	Children; mean = 3.4	6.1%	69.9%	—	12.7%	54.5%
Aguirre 2011^43^	Seasonal influenza A	—	Under 18 years; mean = 5.6	5.0%	—	35.0%	0.9%	—
CCDR 2006^61^	Influenza	—	Under 17 years	6.6%	69.3%	—	12.3%	6.4%
Mitchell 2013^36^	Influenza	During pH1N1	Mean = 46.5	89.6%	76.9%	—	25.7%	—
Mitchell 2013^36^	Influenza	Pre-H1N1	Mean = 66.1	39.8%	65.7%	—	12.6%	—
Mitchell 2013^36^	Influenza	Post-pH1N1	Mean = 69.7	79.9%	75.0%	—	12.6%	—
Wilkinson 2010^40^	Seasonal influenza A or B	—	Mean = 57.0	42.0%	82.0%	—	−2006/07 or 2007/08: 9.0% −2008/09: 30.0%	—
Hassan 2012^39^	Influenza	15-day mortality Hospitalized	Median = 65.0	66.0%	91.0%	—	—	55.0%
McGeer 2009^26^	Influenza	Community-acquired	Mean = 67.0	29.0%	—	—	9.0%	—
McGeer 2009^26^	Influenza	Hospital-acquired	Mean = 67.0	75.0%	—	—	9.0%	—
McGeer 2009^35^	Influenza	ICU admission	Median = 73.0	40.0%	90.0%	—	—	—
McGeer 2012^27^	Influenza	Hospitalized; post-pH1N1	Median = 76.0	72.0%	84.0%	—	19.0%	9.4%
McGeer 2012^27^	Influenza	Hospitalized; pre-pH1N1	Median = 77.0	39.0%	82.0%	—	19.0%	11.0%
McGeer 2007^24^	Influenza	Hospitalized	Adults; mean = 77.2	32.0%	89.0%	—	16.0%	—
McGeer 2007^24^	Influenza	Hospitalized	Under 15 years	—	—	—	1.0%	—
Church 2002^28^	Influenza A	—	Elderly; mean = 82.0	69.2%	53.5%	1.30%	—	—
Bowles 2002^29^	Influenza A/H3N2/Sydney/05/97	LTC	Elderly	—	35.0%	11.0%	—	—
O'Riordan 2010^42^	Seasonal influenza A	Hospitalized	Children; median = 3.3	—	—	—	14.0%	– 10.0%– ICU: 68.0%
Pollock 2012^37^	pH1N1 or ILI	Remote FN region	Mean = 6.1	—	—	16.1%	—	—
Cutler 2009^56^	ILI		Mean = 20.1	—	—	0.0%	—	—

Note: flu, influenza; hosp, hospitalization; FN, First Nations; ICU, intensive care unit; ILI, influenza-like illness, LTC, long-term care; MV, mechanical ventilation; NR, not reported; pH1N1, pandemic influenza A(H1N1)pdm2009.

## Hospitalization and intensive care unit (ICU) admission

Numerous studies conducted in the Canadian setting report on the probability of influenza-related hospitalization (Table 3),^28,29,37,43,56,58,64^ the rate of influenza-related hospitalization in the general or hospital population,^40,47,57,58,65-67^ and the length of hospitalization stay (LOS).^24,34,36,37,41-43,49-54,58,60,61,63^ The probability of hospitalization during non-pandemic influenza seasons was higher in children (35.0%)^43^ than in elderly people (1.3–11.0%).^28,29^ Hospital admission rates for individuals with non-pandemic A/H1N1 influenza ranged from 3.9 to 270-340 hospitalizations per 100,000 population.^40,65-67^ Overall, for non-pandemic influenza seasons, LOS ranged from 2 to 10 d.^24,34,36,42,43,60,61^

In general, the probability of admission to the ICU was higher in adults than children (Table 3). Probability of ICU admission for children ranged from 0.9%^43^ to 26.1%^63^ and probabilities in adults ranged from 4.9%^47^ to 30.0%.^40^ Rates of ICU admission ranged from 0.2 per 100,000 population^39^ to 4.4 per 100,000 population.^57^ There was a wide range of probabilities reported for mechanical ventilation, ranging from 6.2%^60^ to 66.0%^63^ in children, and 9.4%^27^ to 55.0% in adults.^39^

## Influenza and productivity loss

Few studies with data on loss of work, or absence from school, or quality-of-life associated with influenza illness were identified in the literature. In an ILI telephone survey, it was estimated that 2 d of work or school were lost per household.^33^ In another study, it was estimated that during non-pandemic influenza seasons from 1997/98 to 2008/09, on average 14 work hours were lost per influenza patient, which translated to an aggregated 20 work days lost per 100 full-time employees. For pandemic strain, while absenteeism rates were similar to those seen in seasonal influenza, an average of 25 work hours were lost per patient.^68^

## Cost of influenza

Limited data exists for the average cost per case for influenza in Canada.^28^ In one study reported in 2002, the authors compared a population of elderly patients who received rapid virus testing to those who did not during the 1998/99 influenza season. Including costs of drugs, laboratory tests, and hospitalizations, the group who received rapid viral testing had a cost of CAN$673.30, compared with CAN$313.85 in the control group.^28^

## Modeling the cost of influenza vaccination

Between 2002 and end of 2013 a number of health economic influenza vaccine studies from Canada were reported, evaluating cost-effectiveness/cost-utility studies of seasonal TIVs, cost of vaccination strategies in targeted groups, and estimated cost-utility analyses and willingness-to-pay (Table 4).^69-75^

**Table 4.** Summary of economic studies of seasonal influenza vaccination in Canada.

Citation	Study	Comparison	Findings
Fisman 2011^69^	Cost-utility analysis conducted using an age-structured compartmental model (dynamic transmission model)	a) MF59-adjuvanted TIV	• Base case showed that MF59-TIV relative to TIV was cost-effective (ICER=CAN$2,111/QALY) in older adults (≥65 years);
		b) TIV	• The cost of using MF59-TIV was higher than TIV over 10 y (CAN$837.0 million and CAN$730.5 million, respectively) which was offset by reducing the healthcare cost of influenza from CAN$501.76 million with TIV to CAN$473.50 million with MF59-TIV.
Tarride 2012^70^	Cost-utility analysis using a decision tree	a) Trivalent LAIV	• The estimated offset per vaccinated child aged 2–17 y for using LAIV versus TIV was CAN$4.20 in direct costs and CAN$35.34 in societal costs.
		b) TIV	
Sander 2009^71^	Cost-utility analysis using a model that simulates influenza transmission	a) No vaccination during the A(H1N1)pdm09 pandemic	• Vaccination of 30% of the population of Ontario against pandemic A(H1N1)pdm09 was estimated to cost CAN$118 million, which was estimated to have reduced the influenza cases rate by 50% vs. no vaccination.
		b) Mass vaccination achieving 30% vaccine coverage during the A(H1N1)pdm09 pandemic	
Sander 2010^72^	Cost-utility analysis conducted using influenza incidence estimates (pre and post influenza implementation)	a) Ontario's universal influenza immunization program	• Universal vaccination vs target group vaccination estimated to reduce care services cost by 52%, and save CAN$1,134 QALYs per season;
		b) Previous program targeted to the high-risk population	• Universal vaccination vs target group vaccination ICER=CAN$10,797/QALY gained.
Skowronski 2006^73^	Cost-effectiveness analysis conducted using a decision analysis	a) Vaccination of high-risk populations only	• Cost of universal vaccination for infants 6–23 months versus target group vaccination was not cost-saving for the health system or from a societal perspective; In the first year, the cost was CAN$17 per day of illness averted, CAN$230 per physician visit averted, CAN$13,000 per hospitalization averted, CAN$900,000/QALY gained, and CAN$6 million per death averted.
		b) Vaccination of high-risk populations only plus all infants/toddlers aged 6–23 months	
Asgary 2012^74^	Contingent valuation	Determined willingness to pay for access to immediate pandemic A(H1N1)pdm09 influenza vaccine	• Households willing to pay CAN$417.35 for immediate A(H1N1)pdm09 vaccination.
Mercer 2009^75^	Cost-analysis	Determined the most important cost drivers and their economic impact on delivering public health funded influenza vaccines within specified budget	• Most significant cost variables for influenza clinics were labor costs and number of vaccines given per nurse per hour.

Note: ICER, incremental cost-effectiveness ratio; LAIV, intranasal live attenuated influenza vaccine, trivalent; QALY, quality-adjusted life year; QIV, quadrivalent inactivated influenza vaccine; TIV, trivalent inactivated influenza vaccine.

A study of MF59-TIV compared with TIV used an age-structured model in which the population was divided into 5 different compartments or disease states: susceptible, vaccinated, exposed, infectious, and recovered.^69^ The probability of moving between compartments was derived from epidemiological data and calibrated to Canadian influenza data (1997 to 2004 seasons). The simulation allowed new individuals to enter through birth or leave through non-influenza deaths over a 10 y time horizon. In the base case, the cost of using MF59-TIV over 10 y was higher (CAN$837.0 million) than TIV (CAN$730.5 million). These costs were offset by reducing the healthcare cost of influenza from CAN$501.76 million with TIV to CAN$473.50 million with MF59-TIV. The incremental cost per quality-adjusted life year (QALY) gained of MF59-TIV relative to TIV was CAN$2,111 in older adults (≥65 years).^69^ In another modeling study, a decision tree was used to assess the cost-effectiveness of trivalent LAIV vs injectable inactivated TIV in children aged 2–17 y with a 1-year time horizon. The study showed that overall, trivalent LAIV dominated TIV and provided an estimated cost savings per vaccinated child of CAN$4.20 from a healthcare perspective and CAN$35.34 from a societal perspective.^70^

Several studies discuss vaccinating different population sub-groups or regions. A cost-utility analysis of the A(H1N1)pdm09 mass immunization program in Ontario predicted a cost of CAN$118 million (CAN$1,645/QALY gained) to immunize 30% of the provincial population.^71^ In a cost-utility analysis of Ontario's universal vaccination program, universal versus targeted group vaccination predicted a cost of CAN$10,797/QALY gained, which is below the threshold usually considered cost-effective in Canada (CAN$40,000–50,000/QALY gained).^72^ In another study published in 2006, a decision analysis was used to assess the cost-effectiveness of universal vaccination of infants/toddlers aged 6–23 months compared with vaccinating high-risk children only.^73^ Analyses were conducted for a cohort of 500,000 children assuming different vaccination coverage between influenza seasons (100% in year 1, 33% thereafter). In the first year, the incremental cost of vaccination from the healthcare system perspective was CAN$17 per day of illness averted, CAN$900,000/QALY gained, and CAN$6 million per death averted. In subsequent years, costs among those vaccinated would equal those unvaccinated (break-even) at a cost of CAN$6.81 per vaccine dose from a healthcare perspective, and CAN$11.90 per vaccine dose from a societal perspective.^73^

Contingent valuation has been used to determine the willingness-to-pay for access to a pandemic influenza vaccine, and found that households were willing to pay CAN$417.35 for immediate A(H1N1)pdm09 vaccination.^74^ In another analysis to determine key cost drivers in providing influenza vaccines within budget conducted in a health unit in Ontario, costs relating to labor (nursing and clerical), supplies, disposals, facilities, mileage, and program costs for influenza clinics (including labor costs) were estimated. This study reported that the most significant cost variables were labor costs and number of vaccines given per nurse per hour.^75^

## Discussion

Based on previous modeling of US surveillance data across 10 influenza seasons, the public health benefit of switching from TIV to QIV is likely to vary depending upon the epidemic intensity of influenza B during each season.^8^ As such, many countries, including Canada, have established sentinel surveillance networks to help monitor influenza disease and to guide public health decision-making. Using data from the national surveillance system and published studies identified by a comprehensive literature search across 2002–2013, we have described the burden of seasonal influenza disease and the cost-effectiveness of vaccination in Canada, with the aim of developing a dataset that may be used to develop future analytical frameworks to model the effect of QIV in Canada.

According to Canadian national surveillance, influenza A was the dominant subtype circulating in all but 2 seasons (2000/01 and 2011/12), whereas influenza B was less prevalent, representing an average of 17% of all laboratory-confirmed cases during the 2001/02 to 2012/13 influenza seasons.^4^ However, the surveillance data showed that influenza B strains from the Yamagata and Victoria lineages co-circulated in 7 of the 12 influenza seasons, resulting in mismatch with the TIV. Viral circulation and the occurrence of B lineage vaccine mismatch reported in Canada was generally consistent with that observed in Europe and the US.^5,76-78^

Studies in the US and Europe suggest that A/H3N2 is the most common subtype associated with complications and death in older adults, followed by influenza B, and then A/H1N1.^79-81^ In children and young adults, influenza B is suggested to pose the highest risk of severe outcomes.^5,7,82^ As we describe above, laboratory confirmed influenza infection rates in Canada were generally highest among elderly populations, followed by children aged <5 years. These two groups, along with people with medical co-morbidities, are at increased risk for influenza complications and are recommended for annual seasonal vaccination in most industrialized countries.^4^

The WHO reports that the global attack rate of influenza is about 10–20%,^83^ while an analysis based on US hospital discharge records collected for 22 seasons (1979–2001), estimated that 230,000 influenza-related hospital admissions occurred annually.^79^ In a more recent surveillance study of laboratory-confirmed influenza-related hospitalizations between 2005 and 2008 in the US, the annual age-adjusted rate of influenza-related hospitalizations was 12.2 per 100,000 persons.^84^ The rate of hospitalization reported in Canada is consistent with that reported in the US. For example, in an evaluation of influenza-related hospitalization in Toronto in which the median age of patients was about 76–77 years, the rate of hospitalization ranged from 2.6 per 100,000 population in the 2005/06 season to 27.1 per 100,000 population in the 2010/11 season.^27^

We are aware that a number of Canadian studies have been published since the 2013 search cut-off data used to inform this review, some of which we describe below. From an epidemiological perspective, the Serious Outcomes Surveillance Network of the Canadian Immunization Research Network reported an interim analysis for the 2014/15 influenza season. They found that the great majority (99%) of laboratory-confirmed influenza hospitalizations with a known influenza virus type reported in their sentinel hospitals was due to influenza A. Almost 70% of hospitalized laboratory-confirmed influenza cases were >75 years, and 7.9% of all hospitalized cases died in hospital.^85^

From a health economic perspective, a recent systematic review provided a qualitative appraisal of all vaccine economic evaluations in Canada published between 1993 and 2013, including influenza cost-effectiveness studies.^86^ While this review's aim was primarily to evaluate and report on study characteristics rather than outcomes *per se*, those identified for influenza were described either as cost-effective or dominant. Subsequent to the systematic search, 2 further economic evaluations of the benefits of switching from TIV to QIV have been published, one of which used a static model, and the second a dynamic model.^87,88^ The first study used a probabilistic and static cost-utility model adapted for Ontario, and found that, in comparison to TIV, universal vaccination with QIV is estimated to realize reductions of an additional 2,516 influenza cases, 1,683 influenza-associated medical visits, 27 influenza-associated hospitalizations, and 5 influenza-associated deaths. Furthermore, QIV would generate 76 additional QALYs with a net budget impact of CAN$4,784,112 from a societal perspective, and with an incremental cost-effectiveness ratio (ICER) of CAN$63,773/QALY relative to TIV.^87^ The second study used an age-structured compartmental dynamic transmission model applied to the overall Canadian population.^88^ This study found that switching from TIV to QIV across Canada would prevent an estimated 328 (6.8%) deaths; 1,876 (5.7%) hospitalizations; 3,395 (5.7%) ER visits; 52,200 (4.9%) doctor's office visits; and 135,538 (4.6%) symptomatic cases of influenza in an average season. The incremental cost-utility ratio (ICUR) for QIV compared with TIV was CAN$7,961 per QALY gained.^88^

In 2004, the NACI recommended annual influenza vaccination in healthy infants/toddlers aged from 6 to 23 months in addition to the groups already recommended for vaccination and most Canadian provinces adopted the recommendation within their publically-funded immunization programs.^89^ In the 2014/15 influenza season, universal influenza vaccination of healthy individuals from 6 months of age was recommended by NACI in Canada.^4^

The incremental reduction in the burden of influenza B disease associated with switching from TIV to QIV was initially modeled by the US Centers for Disease Control and Prevention (CDC).^8^ It was estimated that this switch would result in fewer influenza cases, hospitalizations, and deaths, dependant on the proportion of cases due to influenza B included in the analysis. As we describe above, modeling studies performed for the Canadian population show similar benefits.^87,88^

We should acknowledge that the present review has some limitations. While we performed a comprehensive systematic search to identify and select studies to inform this review, no formal systematic appraisal of these studies was performed. As such, bias, both in the study selection and in the original research (including reporting and selection bias) may exist, and this may impact upon our summary of the data. It may be noted however, that while reporting bias may exist, some consider these to have a particular impact when discussing vaccine effectiveness (VE) data. In our review, we specifically do not discuss VE data, in part as data is limited for the Canadian population, although we accept that this omission may also be a limitation of this review.

Another limitation was in the search cut-off date. While we have sought to mitigate the latter by making some mention of relevant more recent literature, it remains that other relevant recent literature has not been included in this review. In addition, reporting differences exist between epidemiological data reported in journals and those reported in surveillance reports (‘gray’ literature). The latter, inclusion of which we consider a strength of the review, often report preliminary, aggregated data, with less detail than formal reports and should be considered as complementary to those data reported in formal publications.

In summary, Canadian national surveillance data show that over 11 seasons, influenza A was the predominant influenza virus, and excluding the pandemic seasons, about 26–34% of cases were attributed to influenza B viruses. Published studies suggest that influenza-related complications are an important cause of hospitalization, antibiotic use, and mortality. Economic models indicate that universal vaccination with TIVs is cost-saving in Canada, with more recent models suggesting that switching from TIV to QIV may further reduce the burden of influenza B disease and be cost-effective. Nevertheless, it remains that outcomes data from the Canadian setting is limited and further real-world data is required to help inform health policy decision makers on appropriate influenza vaccination strategies for Canada.

## Trademark disclosure

*Agriflu* and *Fluad* are trademarks of Seqirus.*FluLaval* and *Fluviral* are trademarks of the GSK group of companies.*FluMist* is a trademark of MedImmune, LLC/the AstraZeneca group of companies.*Fluzone* and *Vaxigrip* are trademarks of Sanofi Pasteur.*Influvac* is a trademark of Abbott Biologicals B.V.

## Supplementary Material

Supplementary Figure and Tables

## Abbreviations

CDC: Centers for Disease Control and Prevention
ER: Emergency Room
GP: General practitioner
HIV+: human immunodeficiency virus-positive
ICU: Intensive Care Unit
ICER: incremental cost-effectiveness ratio
ICUR: incremental cost-utility ratio
ILI: influenza-like illness
LAIV: live attenuated influenza vaccine
LOS: length of hospitalization stay
NACI: National Advisory Committee on Immunization
NML: National Microbiology Laboratory
RT-PCR: real-time polymerase chain reaction
PHAC: Public Health Agency of Canada
QALY: quality-adjusted life year
QIV: quadrivalent influenza vaccine
RVDSS: Respiratory Virus Detection Surveillance System
TIV: trivalent influenza vaccine
US: United States
VE: vaccine effectiveness
WHO: World Health Organization
