# Supplementary material for: Review of seasonal influenza in Canada: Burden of disease and the cost-effectiveness of quadrivalent inactivated influenza vaccines
Source: Hum Vaccin Immunother. 2016 Nov 18;13(4):867–76. doi: 10.1080/21645515.2016.1251537 (PMC5404371; doi:10.1080/21645515.2016.1251537)
Supplement: Supplementary Figure and Tables [file khvi-13-04-1251537-s001.zip › KHVI_A_1251537_Supplement/Supplementary Figure 1.docx]

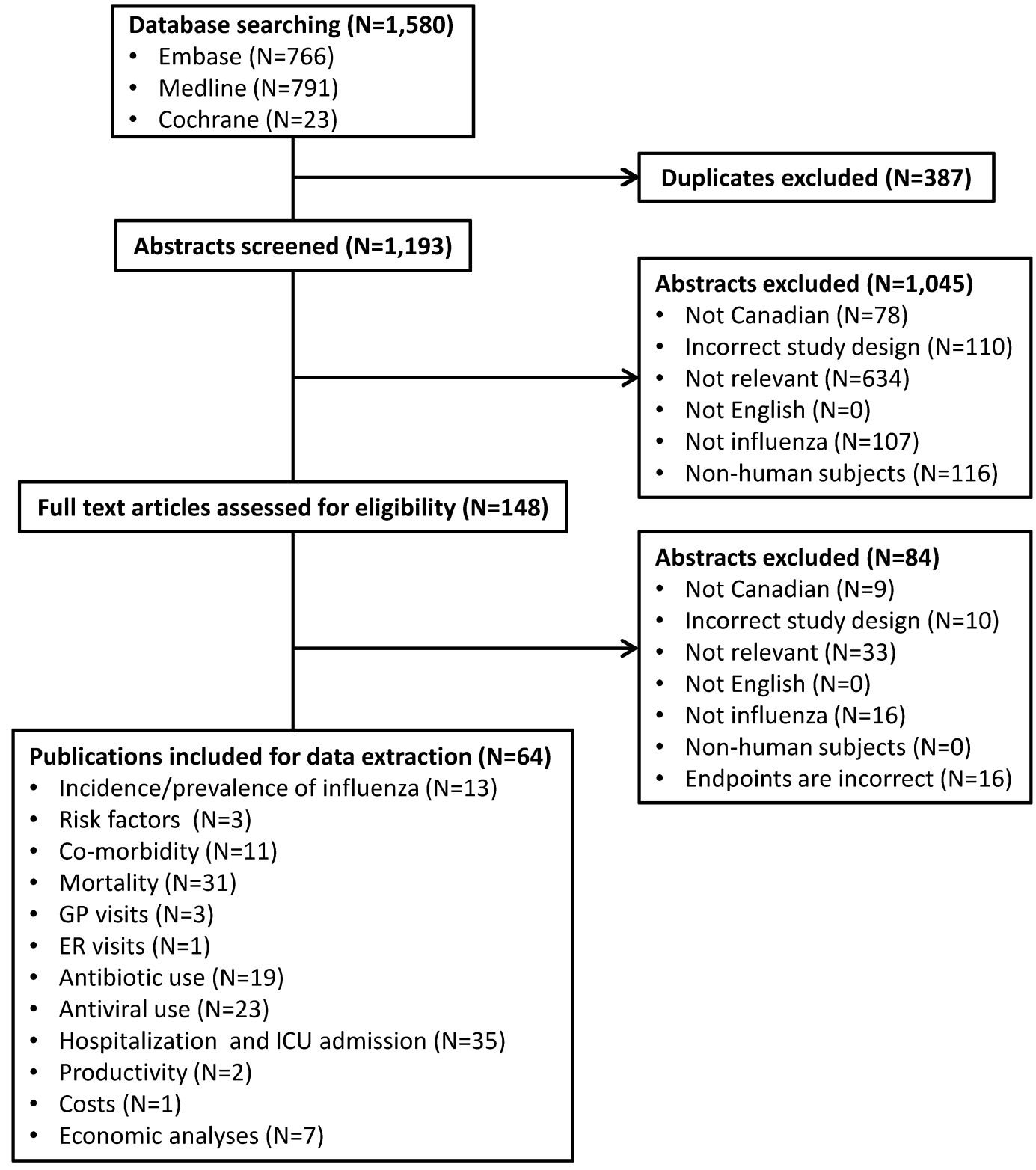


**Supplementary Figure 1. Overview of article selection**

Note: ER, emergency room; GP, general practitioner, ICU, intensive care unit
