# Supplementary material for: Review of seasonal influenza in Canada: Burden of disease and the cost-effectiveness of quadrivalent inactivated influenza vaccines
Source: Hum Vaccin Immunother. 2016 Nov 18;13(4):867–76. doi: 10.1080/21645515.2016.1251537 (PMC5404371; doi:10.1080/21645515.2016.1251537)
Supplement: Supplementary Figure and Tables [file khvi-13-04-1251537-s001.zip › KHVI_A_1251537_Supplement/Supplementary Table 1.docx]

# Supplementary Table 1. Search Strategy: epidemiology

| **PubMed** | | **Number of hits** |
| --- | --- | --- |
| 1 | ((Influenza, Human[MeSH Terms]) OR influenza[Title/Abstract]) OR influenza virus[Title/Abstract] | 71,892 |
| 2 | ((Epidemiology[MeSH Terms]) OR incidence[MeSH Terms]) OR prevalence[MeSH Terms] | 347,322 |
| 3 | (((incidence[Title/Abstract]) OR prevalence[Title/Abstract]) OR epidemiolog*[Title/Abstract]) OR mortality[Title/Abstract] | 1,366,153 |
| 4 | #1 AND (#2 OR #3) | 11,827 |
| 5 | ((((((((((((((canada) OR canadian) OR alberta) OR "british columbia") OR manitoba) OR "new brunswick") OR ("newfoundland and labrador")) OR "northwest territories") OR "nova scotia") OR nunavut) OR ontario) OR "prince edward island") OR quebec) OR saskatchewan) OR "yukon territory" | 566,412 |
| 6 | ((letter[Publication Type]) OR editorial[Publication Type]) OR comment[Publication Type] | 1,280,679 |
| 7 | (#4 AND #5) NOT #6 | 537 |
| 8 | #7 AND Filters: English | 521 |
| 9 | #8 AND Filters: Publications date from 2002/01/01 to 2013/12/31 | 420 |
