# Supplementary material for: Review of seasonal influenza in Canada: Burden of disease and the cost-effectiveness of quadrivalent inactivated influenza vaccines
Source: Hum Vaccin Immunother. 2016 Nov 18;13(4):867–76. doi: 10.1080/21645515.2016.1251537 (PMC5404371; doi:10.1080/21645515.2016.1251537)
Supplement: Supplementary Figure and Tables [file khvi-13-04-1251537-s001.zip › KHVI_A_1251537_Supplement/Supplementary Table 3.docx]

**Supplementary Table 3. Search strategy: quality of life and economic analyses**

| **PubMed** | | **Number of hits** |
| --- | --- | --- |
| 1 | ((Influenza, Human[MeSH Terms]) OR influenza[Title/Abstract]) OR influenza virus[Title/Abstract] | 71,892 |
| 2 | (("quality of life"[MeSH Terms]) OR "health status indicators"[MeSH Terms]) OR "models, economic"[MeSH Terms] | 288,266 |
| 3 | (((((((((("quality of life"[Title/Abstract]) OR qol[Title/Abstract]) OR "quality adjusted life year"[Title/Abstract]) OR qaly[Title/Abstract]) OR utilit*[Title/Abstract]) OR "disability adjusted life"[Title/Abstract]) OR daly*[Title/Abstract]) OR (sf36 OR "sf 36" OR "short form 36" OR "shortform 36"[Title/Abstract])) OR ("sf thirtysix" OR "sf thirty six" OR "shortform thirtysix" OR "shortform thirty six"[Title/Abstract])) OR ("short form thirtysix" OR "short form thirty"[Title/Abstract])) OR (sf6 OR "sf 6" OR "short form 6" OR "shortform 6" OR "sf six" OR sfsix OR "shortform six" OR "short form six") | 264,435 |
| 4 | (((((sf12 OR "sf 12" OR "short form 12" OR "shortform 12" OR "sf twelve" OR sftwelve OR "shortform twelve" OR "short form twelve"[Title/Abstract])) OR (sf16 OR “sf 16” OR “short form 16” OR “shortform 16” OR “sf sixteen” OR sfsixteen OR “shortform sixteen” OR “short form sixteen”[Title/Abstract])) OR (sf20 OR “sf 20” OR “short form 20” OR “shortform 20” OR “sf twenty” OR sftwenty OR “shortform twenty” OR “short form twenty”[Title/Abstract])) OR (euroqol OR "euro qol" OR eq5d OR "eq 5d"[Title/Abstract])) OR (hql OR hqol OR "h qol" OR hrqol OR "hr qol" OR hye or hyes[Title/Abstract]) | 17,848 |
| 5 | (((((((((((((health* year* equivalent*[Title/Abstract]) OR health utilit*[Title/Abstract]) OR (hui OR hui1 OR hui2 OR hui3[Title/Abstract])) OR disutili*[Title/Abstract]) OR rosser[Title/Abstract]) OR "quality of wellbeing"[Title/Abstract]) OR qwb[Title/Abstract]) OR "willingness to pay"[Title/Abstract]) OR standard gamble*[Title/Abstract]) OR ("time trade off" OR "time tradeoff"[Title/Abstract])) OR tto[Title/Abstract]) OR economic model*[Title/Abstract]) OR markov*[Title/Abstract]) OR "monte carlo"[Title/Abstract] | 63,488 |
| 6 | (((("decision tree"[Title/Abstract]) OR "decision analysis"[Title/Abstract]) OR "decision analyses"[Title/Abstract]) OR "decision model"[Title/Abstract]) OR "decision models"[Title/Abstract] | 7,632 |
| 7 | (((((((((((((pharmacoeconomic*[Title/Abstract]) OR "health economic"[Title/Abstract]) OR "economic evaluation"[Title/Abstract]) OR "economic model"[Title/Abstract]) OR "economic models"[Title/Abstract]) OR "economic analysis"[Title/Abstract]) OR "decision analytic model"[Title/Abstract]) OR cost-effectiveness[Title/Abstract]) OR cost-benefit[Title/Abstract]) OR cost-utility[Title/Abstract]) OR cost-minimisation[Title/Abstract]) OR cost-minimization[Title/Abstract]) OR budget[Title/Abstract]) OR "budget impact"[Title/Abstract] | 60,904 |
| 8 | #1 AND (#2 OR #3 OR #4 OR #5 OR #6 OR #7) | 2,000 |
| 9 | ((((((((((((((canada) OR canadian) OR alberta) OR "british columbia") OR manitoba) OR "new brunswick") OR ("newfoundland and labrador")) OR "northwest territories") OR "nova scotia") OR nunavut) OR ontario) OR "prince edward island") OR quebec) OR saskatchewan) OR "yukon territory" | 566,412 |
| 10 | ((letter[Publication Type]) OR editorial[Publication Type]) OR comment[Publication Type] | 1,280,679 |
| 11 | (#8 AND #9) NOT #10 | 131 |
| 12 | #11 AND Filters: English | 129 |
| 13 | #12 AND Filters: Publications date from 2002/01/01 to 2013/12/31 | 112 |
