# Supplementary material for: Review of seasonal influenza in Canada: Burden of disease and the cost-effectiveness of quadrivalent inactivated influenza vaccines
Source: Hum Vaccin Immunother. 2016 Nov 18;13(4):867–76. doi: 10.1080/21645515.2016.1251537 (PMC5404371; doi:10.1080/21645515.2016.1251537)
Supplement: Supplementary Figure and Tables [file khvi-13-04-1251537-s001.zip › KHVI_A_1251537_Supplement/Supplementary Table 4.docx]

**Supplementary Table 4. Studies reporting the incidence of seasonal influenza in Canada**

| **Source** | **Disease** | **Setting** | **Age (years)** | **Sample** | **Rate** |
| --- | --- | --- | --- | --- | --- |
| McGeer 2007^24^ | Influenza | Hospitalized  2006-2007 | Children under 15 years  Adults Mean=77.2 | 327 confirmed adult cases,  185 confirmed child cases | 0.44 per 1,000 patients |
|  |  | Hospitalized  2007-2008 | Children under 15 years  Adults Mean=77.2 |  | 1.0 per 1,000 patients |
| Schull 2005^25^ | Influenza A and B | ED visits 1996 | Mean=43.3 | 11,075 Mean weekly total ED visits | 66 per 1,000 patients |
|  |  | ED visits 1997 | Mean=43.3 |  | 164 per 1,000 patients |
|  |  | ED visits 1998 | Mean=43.3 |  | 601 per 1,000 patients |
|  |  | ED visits 1999 | Mean=43.3 |  | 595 per 1,000 patients |
| McGeer 2009^26^ | Influenza | Hospitalized  2006–2007 | Mean=67 | Patients in 15 hospitals from CNISP network | 1.1 per 1,000 patients |
|  |  | Hospitalized  2007–2008 | Mean=67 | Patients in 11 hospitals from CNISP network | 2.7 per 1,000 patients |
| McGeer 2012^27^ | Hospitalized influenza | General population  2004–2005 | Pre-pH1N1 Median=77  Post-pH1N1 Median=76 | 4 million population in metro Toronto/Peel region | 9.3 per 100,000 population |
|  |  | General population  2005–2006 | Pre-pH1N1 Median=77  Post-pH1N1 Median=76 |  | 2.6 per 100,000 population |
|  |  | General population  2006–2007 | Pre-pH1N1 Median=77  Post-pH1N1 Median=76 |  | 5.3 per 100,000 population |
|  |  | General population  2007–2008 | Pre-pH1N1 Median=77  Post-pH1N1 Median=76 |  | 14.3 per 100,000 population |
|  |  | General population  2008–2009 | Pre-pH1N1 Median=77  Post-pH1N1 Median=76 |  | 2.9 per 100,000 population |
|  |  | General population  2010–2011 | Pre-pH1N1 Median=77  Post-pH1N1 Median=76 |  | 27.1 per 100,000 population |
| Church 2002^28^ | Influenza A | Nursing homes | Elderly; mean=82.0 | 1,705 nursing home residents | 17,600 per 100,000 population |
| Bowles 2002^29^ | Influenza A/H3N2/Sydney/05/97 | LCF | Elderly | 993 residents in 10 LCFs | 5,000-43,000 per 100,000 population |
| Mahmud 2013^30^ | Influenza A | LCF | Staff | 154 ILI outbreaks in 39 LCFs in the Winnipeg Health Region | 3,500 per 100,000 population |
|  | Influenza B | LCF | Staff |  | 3,100 per 100,000 population |
|  | Influenza A | LCF | Elderly |  | 7,400 per 100,000 population |
|  | Influenza B | LCF | Elderly |  | 6,400 per 100,000 population |
| Kuster 2010^31^ | Influenza A | Hospitalized  2007–2008 | -- | 6,236 hospital admissions | 21.8 per 1,000 patients |
|  | Influenza B | Hospitalized  2007–2008 | -- |  | 23 per 1,000 patients |
| Janjua 2012^32^ | ILI | Telephone survey 2009 pandemic | Median=15 | 408 students | 22,549 per 100,000 population |
| Ammons 2009^33^ | ILI | Telephone survey | -- | 1,009 households | 27,000 per 100,000 population |

Note: CNISP, the Canadian Nosocomial Infection Surveillance Program; ED, emergency department; ILI, influenza-like illness; LCF, Long-term Care Facility; pH1N1, pandemic influenza A(H1N1)pdm2009
