# Supplementary material for: Review of seasonal influenza in Canada: Burden of disease and the cost-effectiveness of quadrivalent inactivated influenza vaccines
Source: Hum Vaccin Immunother. 2016 Nov 18;13(4):867–76. doi: 10.1080/21645515.2016.1251537 (PMC5404371; doi:10.1080/21645515.2016.1251537)
Supplement: Supplementary Figure and Tables [file khvi-13-04-1251537-s001.zip › KHVI_A_1251537_Supplement/Supplementary Table 5.docx]

**Supplementary Table 5. Studies reporting risk factors and co-morbidities in populations with seasonal influenza in Canada**

| **Source** | **Disease** | **Setting** | **Age (years)** | **Risk factors** | **Odds ratio (95% CI)** |
| --- | --- | --- | --- | --- | --- |
| Tran 2012^34^ | Seasonal influenza A | Sep 2004–Mar 2010 | Mean=3.4 | Received seasonal influenza vaccine | 0.48 (0.26–0.88) |
|  |  |  |  | Neurologic condition | 1.90 (1.22–2.95) |
| Janjua 2012^32^ | ILI | May 2009–June 2009 | Median=15 | Receipt of 2008–2009 TIV | 2.38 (1.26-4.5) |
|  |  |  |  | Presence of co-morbidity | 2.65 (1.16–6.05) |
|  |  |  |  | Younger age 1–8 years | 5.2 (2.61–10.36) |

| **Source** | **Disease** | **Setting** | **Age (years)** | **Co-morbidity** | **Proportion of cases [% (n)]** |
| --- | --- | --- | --- | --- | --- |
| Tran 2012^34^ | Seasonal influenza A | Hospitalized | Mean=5.7 | Pneumonia | 34.1% |
|  |  |  |  | Croup | 7.2% |
|  |  |  |  | Encephalitis | 6% |
|  |  |  |  | Myocarditis or Cardiomyopathy | 1.8% |
|  |  |  |  | Myositis | 0.6% |
|  |  |  |  | Hepatitis | 0.6% |
| McGeer 2007^24^ | Seasonal influenza | Hospitalized | Children under 15 | Asthma | 25% |
| Bowles 2002^29^ | Influenza A/H3N2/Sydney/05/97 | LCF | Elderly | Heart disease | 61% had at least one of the co-morbidities |
|  |  |  |  | Lung disease |  |
|  |  |  |  | Diabetes |  |
|  |  |  |  | Active Malignancy |  |
|  |  |  |  | Cerebrovascular disease |  |
| McGeer 2009^26^ | Influenza | Hospitalized | Mean=67 | Pneumonia | --  --  --  -- |
|  |  |  |  | Exacerbation of COPD |  |
|  |  |  |  | Sepsis or fever |  |
|  |  |  |  | Cardiac diagnoses |  |
| McGeer 2009^35^ | Influenza | ICU admission | Median=73.0 | Pneumonia | 61% |
|  |  |  |  | Cardio-respiratory diagnosis | 20% |
|  |  |  |  | Bacteremia | 9% |
| McGeer 2012^27^ | Influenza | Hospitalized | - Pre-pH1N1 Median=77 - Post-pH1N1 Median=76 | Pneumonia | 37% |
| McGeer 2007^24^ | Seasonal influenza | Hospitalized | Adults Mean=77.2 | Chronic illness | 75% |
|  |  |  |  | Cardiac disease | 42% |
|  |  |  |  | Pulmonary disease | 34% |
|  |  |  |  | Diabetes | 28% |
|  |  |  |  | Cancer | 10% |
|  |  |  |  | Renal disease | 9.2% |
| Mitchell 2013^36^ | Influenza | Community-acquired; pre-H1N1 | Mean=66.1 | Any complication | 70.9% |
|  |  |  |  | Chronic lung disease | 21.8% |
|  |  |  |  | Chronic heart disease | 18.3% |
|  |  |  |  | Diabetes | 14.1% |
|  |  |  |  | Immune suppressed | 11.8% |
|  |  |  |  | Chronic kidney disease | 7.2% |
| Mitchell 2013^36^ | Influenza | Community-acquired; post-H1N1 | Mean=69.7 | Any complication | 86.4% |
|  |  |  |  | Chronic heart disease | 35.4% |
|  |  |  |  | Chronic lung disease | 30.4% |
|  |  |  |  | Diabetes | 21.8% |
|  |  |  |  | Immune suppressed | 16.6% |
|  |  |  |  | Chronic kidney disease | 11.2% |
| Pollock 2012^37^ | pH1N1 or ILI | Remote FN region | Median=1.9 | Cough | 96.1% |
|  |  |  |  | Fever | 72.1% |
|  |  |  |  | Anorexia | 51.4% |
|  |  |  |  | Vomiting | 50.8% |
|  |  |  |  | Rhinorrhoea | 35.2% |
|  |  |  |  | Diarrhoea | 26.3% |
|  |  |  |  | Earache | 22.3% |
|  |  |  |  | Sore throat | 21.2% |

Note: CI, confidence interval; COPD, chronic obstructive pulmonary disease; FN, First Nations; ICU, intensive care unit; ILI, influenza-like illness; LCF, Long-term Care Facility; pH1N1, pandemic influenza A(H1N1)pdm2009; TIV, trivalent influenza vaccine
