# Supplementary material for: Review of seasonal influenza in Canada: Burden of disease and the cost-effectiveness of quadrivalent inactivated influenza vaccines
Source: Hum Vaccin Immunother. 2016 Nov 18;13(4):867–76. doi: 10.1080/21645515.2016.1251537 (PMC5404371; doi:10.1080/21645515.2016.1251537)
Supplement: Supplementary Figure and Tables [file khvi-13-04-1251537-s001.zip › KHVI_A_1251537_Supplement/Supplementary Table 6.docx]

**Supplementary Table 6. Summary of studies reporting the rate and probability of influenza-related death**

| **Source** | **Disease** | **Population** | **Age (years)** | **p(death\|flu)** |
| --- | --- | --- | --- | --- |
| Moore 2006^60^ | Influenza | -- | Children; median=1.7 | 0.6% |
| O'Riordan 2010^42^ | Seasonal influenza A | Hospitalized | Children; median=3.3 | 0.5% |
| Tran 2012^34^ | Seasonal influenza A | Hospitalized | Children; mean=3.4 | 0.5% |
| Aguirre 2011^43^ | Seasonal influenza A | -- | Under 18 years; mean=5.6 | 0% |
| McGeer 2007^24^ | Influenza | 15-day mortality | Under 15 years | 0.0% |
| CCDR 2006^61^ | Influenza | -- | Under 17 years | 0.5% |
| Mitchell 2013^36^ | Influenza | Overall; during pH1N1 | Mean=46.5 | 6.4% |
| Mitchell 2013^36^ | Influenza | Direct-attributable; during H1N1 | Mean=46.5 | 5.5% |
| Wilkinson 2010^40^ | Seasonal influenza A or B | 30-day mortality, all cause; 2007-08 | Mean=57.0 | 8.0% |
| Wilkinson 2010^40^ | Seasonal influenza A or B | Direct-attributable; 2008-09 | Mean=57.0 | 2.8% |
| Wilkinson 2010^40^ | Seasonal influenza A or B | 30-day mortality, all cause; 2007-08 | Mean=57.0 | 11.0% |
| Wilkinson 2010^40^ | Seasonal influenza A or B | Direct-attributable; 2008-09 | Mean=57.0 | 8.0% |
| Hassan 2012^39^ | Influenza | 15-day mortality | Median=65.0 | 19% |
| Hassan 2012^39^ | Influenza | Hospitalized | Median=65.0 | 27% |
| Mitchell 2013^36^ | Influenza | Overall; pre-H1N1 | Mean=66.1 | 8.4% |
| Mitchell 2013^36^ | Influenza | Direct-attributable; pre-H1N1 | Mean=66.1 | 4.0% |
| McGeer 2009^35^ | Influenza | 30-day mortality; direct-attributable | Mean=67.0 | 2.6% |
| Mitchell 2013^36^ | Influenza | Overall; post-pH1N1 | Mean=69.7 | 6.7% |
| Mitchell 2013^36^ | Influenza | Direct-attributable; post-H1N1 | Mean=69.7 | 4.5% |
| McGeer 2009^35^ | Influenza | ICU admission | Median=73.0 | 25.0% |
| McGeer 2012^27^ | Influenza | 15-day mortality; post-pH1N1 | Median=76.0 | 7.4% |
| McGeer 2012^27^ | Influenza | 15-day mortality; pre-pH1N1 | Median=77.0 | 8.6% |
| McGeer 2007^24^ | Influenza | 15-day mortality | Adults; mean=77.2 | 8.3% |
| Church 2002^28^ | Influenza A | -- | Elderly; mean=82.0 | 8.80% |
| Bowles 2002^29^ | Influenza A/H3N2/Sydney/05/97 | LTC | Elderly | 9% |
| Wilkinson 2010^59^ | Influenza | 30-day mortality, all cause | NR | 5.0% |
| Wilkinson 2010^59^ | Influenza | Direct-attributable | NR | 4.4% |
| Jouvet 2010^41^ | pH1N1 | PICU admission | Under 18 years; median=5.0 | 7% |
| Tran 2012^34^ | pH1N1, 1st wave | Hospitalized | Children, mean=6.3 | 0.9% |
| O'Riordan 2010^42^ | pH1N1 | Hospitalized | Children; median=6.4 | 0.0% |
| Aguirre 2011^43^ | pH1N1 | -- | Under 18 years; mean=9.5 | 0% |
| Flechelles 2013^44^ | pH1N1, 1st wave | -- | Children | 7% |
| WHO 2009^58^ | pH1N1 | -- | Median=16.0 | 0.2% (Rate: 0.02 per 100,000 population) |
| Allard 2010^45^ | pH1N1 | Hospitalized | Mean=28.6 | 4.32% |
| Kumar 2009^46^ | pH1N1 | -- | Mean=32.3 | 17.3% |
| Kumar 2009^46^ | pH1N1 | 14-day mortality | Mean=32.3 | 10.7% |
| Kumar 2009^46^ | pH1N1 | 28-day mortality | Mean=32.3 | 14.3% |
| Campbell 2010^47^ | pH1N1 | Hospitalized | Mean=34.8 | 4.9% |
| Campbell 2010^47^ | pH1N1 | ICU admission | Mean=34.8 | -- (Rate: 0.21 per 100,000 population) |
| Sood 2010^48^ | pH1N1 | ICU admission | Mean=35.5 | 16.0% |
| Jung 2011^49^ | pH1N1 | Hospitalized; aboriginal | Mean=41.0 | 15.6% |
| Gu 2011^50^ | pH1N1, 1st wave | Hospitalized | Mean=44.7 | 19.6% |
| Muscedere 2011^52^ | pH1N1 | ICU admission | Mean=46.7 | 22.8% |
| McNeil 2010^51^ | pH1N1 | -- | Mean=47.0 | 6.0% |
| Gu 2011^50^ | pH1N1, 2nd wave | Hospitalized | Mean=47.7 | 25.6% |
| Muscedere 2013^53^ | pH1N1 | ICU admission | Mean=47.9 | 21.7% |
| Bagshaw 2013^54^ | pH1N1 | ICU admission | Mean=48.0 | 20.7% |
| Jung 2011^49^ | pH1N1 | Hospitalized; non-aboriginal | Mean=48.0 | 23.8% |
| Taylor 2011^55^ | pH1N1 | 30-day mortality, all cause | Median=49 | 7.0% |
| Taylor 2011^55^ | pH1N1 | Direct-attributable | Median=49 | 6.0% |
| Helferty 2010^57^ | pH1N1, 1st wave | -- | Median=23.0 | -- (Rate: 0.2 per 100,000 population) |
| Helferty 2010^57^ | pH1N1, 2nd wave | -- | Median=30.0 | -- (Rate: 1.0 per 100,000 population) |
| Helferty 2010^57^ | pH1N1, both waves | -- | NR | -- (Rate: 1.3 per 100,000 population) |
| Cutler 2009^56^ | ILI | -- | Mean=20.1 | 0% |

Note: ICU, intensive care unit; ILI, influenza like illness; LTC, long-term care; NR, not reported; pH1N1, pandemic H1N1; PICU, pediatric intensive care unit

Population refers to any characteristic in the study that could lead to a higher (or lower) risk of influenza resource use; within disease category, studies are sorted on mean/median age, where available
